# FOXP3+ Regulatory T Cell Compartment Is Altered in Children With Newly Diagnosed Type 1 Diabetes but Not in Autoantibody-Positive at-Risk Children

**DOI:** 10.3389/fimmu.2019.00019

**Published:** 2019-01-22

**Authors:** Tyyne Viisanen, Ahmad M. Gazali, Emmi-Leena Ihantola, Ilse Ekman, Kirsti Näntö-Salonen, Riitta Veijola, Jorma Toppari, Mikael Knip, Jorma Ilonen, Tuure Kinnunen

**Affiliations:** ^1^Department of Clinical Microbiology, Institute of Clinical Medicine, University of Eastern Finland, Kuopio, Finland; ^2^Department of Pediatrics, Turku University Hospital, Turku, Finland; ^3^PEDEGO Research Unit, Department of Pediatrics, Medical Research Center, Oulu University Hospital and University of Oulu, Oulu, Finland; ^4^Department of Physiology, Institute of Biomedicine, University of Turku, Turku, Finland; ^5^Tampere Center for Child Health Research, Tampere University Hospital, Tampere, Finland; ^6^Children's Hospital, University of Helsinki and Helsinki University Hospital, Helsinki, Finland; ^7^Research Programs Unit, Diabetes and Obesity, University of Helsinki, Helsinki, Finland; ^8^Folkhälsan Research Center, Helsinki, Finland; ^9^Immunogenetics Laboratory, Institute of Biomedicine, University of Turku, Turku, Finland; ^10^Clinical Microbiology, Turku University Hospital, Turku, Finland; ^11^Eastern Finland Laboratory Centre (ISLAB), Kuopio, Finland

**Keywords:** autoimmunity, human, type 1 diabetes, immune regulation, T cells, regulatory T cell, immunophenotyping

## Abstract

The dysfunction of FOXP3-positive regulatory T cells (Tregs) plays a key role in the pathogenesis of autoimmune diseases, including type 1 diabetes (T1D). However, previous studies analyzing the peripheral blood Treg compartment in patients with T1D have yielded partially conflicting results. Moreover, the phenotypic complexity of peripheral blood Tregs during the development of human T1D has not been comprehensively analyzed. Here, we used multi-color flow cytometry to analyze the frequency of distinct Treg subsets in blood samples from a large cohort comprising of 74 children with newly diagnosed T1D, 76 autoantibody-positive children at-risk for T1D and 180 age- and HLA-matched control children. The frequency of CD4+CD25+CD127lowFOXP3+ Tregs was higher in children with T1D compared to control children, and this change was attributable to a higher proportion of naïve Tregs in these subjects. Further longitudinal analyses demonstrated that the increase in Treg frequency correlated with disease onset. The frequencies of the minor subsets of CD25+FOXP3low memory Tregs as well as CD25lowCD127lowFOXP3+ Tregs were also increased in children with T1D. Moreover, the ratio of CCR6-CXCR3+ and CCR6+CXCR3- memory Tregs was altered and the frequency of proliferating Ki67-positive and IFN-γ producing memory Tregs was decreased in children with T1D. The frequency of CXCR5+FOXP3+ circulating follicular T regulatory cells was not altered in children with T1D. Importantly, none of the alterations observed in children with T1D were observed in autoantibody-positive at-risk children. In conclusion, our study reveals multiple alterations in the peripheral blood Treg compartment at the diagnosis of T1D that appear not to be features of early islet autoimmunity.

## Introduction

Type 1 diabetes (T1D) is an autoimmune disease characterized by a T-cell-mediated destruction of insulin-producing β-cells in the pancreas (1). In humans, the diagnosis of T1D is typically preceded by a period of asymptomatic autoimmunity characterized by the presence of islet autoantibodies, such as insulin autoantibodies (IAA) and antibodies against GAD (GADA), islet antigen 2 (IA-2A) and zinc transporter 8 (ZnT8A), that are highly predictive of future disease (2, 3).

CD4+FOXP3+ regulatory T cells (Tregs) are a specialized subset of helper T cells that have a crucial role in preventing autoimmunity in murine models, including the NOD mouse model for T1D (4–6). In humans, the strongest evidence linking Treg dysfunction and autoimmunity comes from patients with the immunodysregulation polyendocrinopathy enteropathy X-linked (IPEX) syndrome that have loss-of-function mutations in the *FOXP3* gene (7). These patients develop a wide range of autoimmune disorders, including T1D, at a very young age (8). Moreover, among the T1D susceptibility loci identified by genome-wide association studies, several are likely to affect molecules associated with Treg function (e.g., *IL2RA, IL2, PTPN2, CTLA4, IL10*) (9).

Multiple studies have set out to address the potential dysregulation of Tregs in patients with T1D by analyzing whether the frequency of Tregs is altered in peripheral blood. Although some have reported both increased (10, 11) and decreased (12) frequencies of Tregs, the majority of studies have concluded that no differences in peripheral blood Treg frequencies exist (13–19). It is, however, noteworthy that several of these studies have used variable markers to define Tregs, and only some have used the most specific markers, CD25 in combination with CD127, FOXP3, and HELIOS (20–23), to define peripheral blood Tregs. Furthermore, in many studies, the patients analyzed have had variable disease duration and were compared to healthy controls that were not stringently matched for age and HLA background. Most studies have also analyzed a rather limited number of individuals (typically < 30 per group), which, given the large interindividual variation in Treg frequencies, reduces the power to detect subtle changes in Treg frequencies. Finally, the data on Treg frequencies during the preclinical phase of T1D is virtually non-existent.

In recent years it has become increasingly clear that peripheral blood FOXP3+ Treg cells are not a uniform population, but rather a heterogeneous mixture of cells of different states of maturation, differentiation and homing capabilities. A recent study employing mass cytometry identified more than 20 distinct subpopulations within the FOXP3+ Treg compartment (24). The seminal study by Miyara et al. demonstrated that the expression of CD45RA (or CD45RO) delineates Tregs into naive, resting Tregs and antigen-experienced memory Tregs (25). The memory Treg compartment can be further subdivided into activated FOXP3hi Tregs that are highly suppressive, and into FOXP3lo Tregs that are poorly suppressive and contain T cells capable of producing proinflammatory cytokines (25). CD39 expression has been shown to identify highly suppressive Tregs that preferentially inhibit Th17-type responses (26, 27). Furthermore, the expression of chemokine receptors, such as CXCR3, CCR6, and CXCR5 appears to identify subpopulations of phenotypically polarized memory Tregs that may be able to selectively suppress their effector T cell counterparts, Th1, Th17 and follicular T helper (Tfh) cells, respectively (28–32). Lastly, a subset of memory Tregs express CD161 which identifies cells capable of producing proinflammatory cytokines, such as IFN-γ and IL-17A (33, 34). Importantly, the few studies that have analyzed the above-mentioned Treg subsets more closely in human T1D have reported subtle changes, such as an increased frequency of naive Tregs (18), FOXP3lo memory Tregs (10) and memory Tregs capable of producing IFN-γ or IL-17A (10, 17).

In the present study, we revisited the question of whether peripheral blood Tregs are altered during the development of human T1D. To this aim, we analyzed Treg frequencies and phenotypic heterogeneity using multi-color flow cytometry and utilizing samples from a large, well-stratified clinical cohort comprising of children with newly diagnosed T1D, autoantibody-positive at-risk children and healthy age- and HLA-matched controls. We observed multiple changes in peripheral blood Treg subsets in children with newly diagnosed T1D but none in autoantibody-positive at-risk children, suggesting that deviation of the peripheral blood Treg compartment is associated with progression to clinical disease rather than being a feature of earlier stages of T1D-associated autoimmunity.

## Materials and Methods

### Study Subjects

The study cohort comprised 74 children with newly diagnosed T1D (< 1 week after clinical diagnosis; mean age 7.8 years ± SD 4.1, age range 1–17 years), 76 auto antibody-positive at-risk children (mean age 9.4 years ± SD 4.7, age range 1–17 years), and 180 autoantibody-negative healthy children (mean age 8.8 years ± SD 4.0, age range 1–16 years). Blood samples for the study were collected between November 2013 and February 2017. With the exception of children with newly diagnosed T1D, all study subjects, including the autoantibody-negative healthy control children, participated in the Finnish Type 1 Diabetes Prediction and Prevention Project (DIPP) follow-up study and had HLA genotypes associated with increased risk for T1D. Autoantibody-positivity was analyzed in the subjects at sampling, as previously described (2). Autoantibody-positive at-risk subjects were defined based on positivity for one or more biochemical autoantibodies (IAA, IA-2A, and/or GADA). Subjects positive for GADA only were excluded from the analyses, since these individuals have a relatively low risk for the development of T1D (2). The study was approved by local ethics committees in the participating university hospitals. All families participating in the study provided written informed consent, as mandated by the Declaration of Helsinki.

### Peripheral Blood Mononuclear Cells Sample Preparation

Peripheral blood mononuclear cells (PBMCs) were isolated from peripheral blood samples by Ficoll gradient centrifugation, resuspended in RPMI 1640 complete medium with 5% human AB serum, and shipped overnight at +4°C from the DIPP study center in Turku to the University of Eastern Finland in Kuopio. Blood samples from healthy age-matched control children were in most cases drawn on the same day and processed in parallel with those from children with newly diagnosed T1D and autoantibody-positive children, allowing us to control for spurious results caused by differential sample preparation through pairwise statistical testing. The viability of the PBMCs before flow cytometric assays was routinely >97%, as assessed by viability staining.

### Flow Cytometric Analyses

Immunostaining for surface markers was performed on 10^6^ PBMCs per staining by incubating the cells with a panel of fluorochrome-labeled antibodies (Supplementary Table 1) for 20 to 30 min. For the determination of cytokine production, PBMCs were first stimulated for 4 h with 20 ng/mL phorbol myristic acid (PMA; Sigma-Aldrich), 500 ng/mL ionomycin (Sigma-Aldrich), and 2 μM monensin (Ebioscience). Fixation and permeabilization were performed using the Foxp3/Transcription Factor Staining Buffer set (eBioscience), followed by staining for intracellular cytokines and transcription factors. The samples were acquired on a FACSCanto II flow cytometer (BD Biosciences), and the flow cytometry data were analyzed using FlowJo software (FlowJo). Coded samples were used throughout, and the flow cytometric analyses were performed blinded to the clinical classification of the sample.

### CXCL10 ELISA

Soluble CXCL10 plasma concentrations were determined using the Human CXCL10/IP-10 Quantikine ELISA Kit (R&D Systems).

### Statistical Analyses

Statistical analyses were performed using Prism software (GraphPad). When comparing differences between multiple groups, one-way ANOVA with Dunnett posttest to correct for multiple comparisons was used. Paired Student *t* tests were used when analyzing paired samples. Relationships between different parameters were examined using Pearson correlation coefficient. *P* < 0.05 was considered to indicate statistical significance.

## Results

### The Frequency of CD4+CD25+CD127lowCD45RA+ Naive Tregs Is Increased in Children With Newly Diagnosed T1D

**Figure 1:**
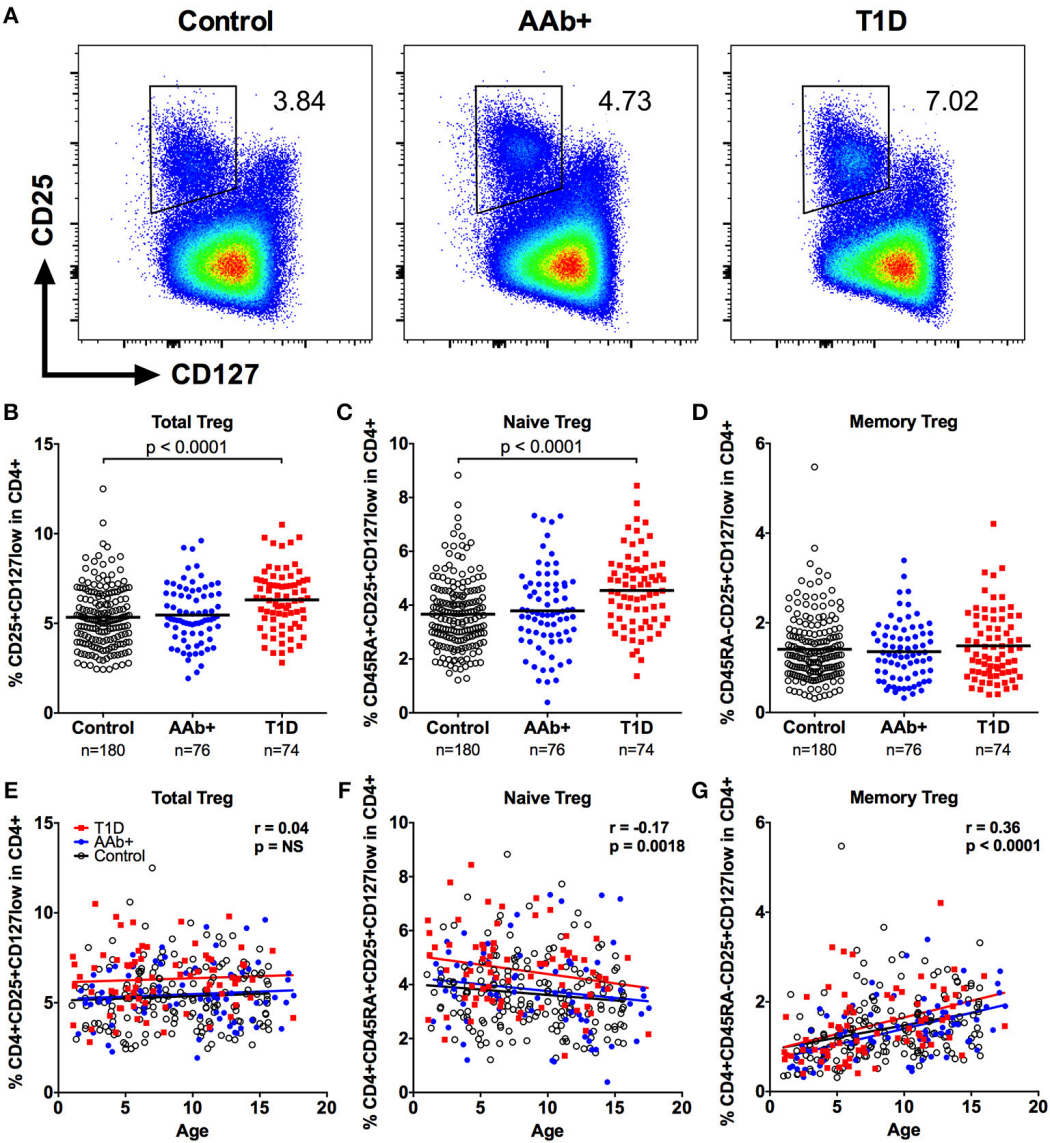
Increased frequency of CD4+CD25+CD127low Tregs in children with newly diagnosed T1D. Representative examples of Treg stainings from healthy control children, autoantibody-positive at-risk children (AAb+) and children with newly diagnosed T1D **(A)**. Frequencies of total **(B)**, naive **(C)**, and memory **(D)** Tregs in control, AAb+ and T1D groups. Linear regression lines for total **(E)**, naive **(F)**, and memory **(G)** Treg frequencies against age were calculated for the control (black lines), AAb+ (blue lines) and T1D (red lines) groups. The elevations of the regression lines were significantly different between the groups for total and naive Tregs (*P* < 0.0001). Correlation with age was calculated by pooling all samples analyzed and is expressed together with *P* values next to the individual plots.

We first examined the frequencies of CD4+CD25+CD127low total Tregs, as well as CD45RA+ naive and CD45RA- memory Treg subsets within the CD4+ T-cell compartment in a large pediatric cohort (Figure 1A and Supplementary Figure 1). In total, peripheral blood samples from 74 children with newly diagnosed T1D, 76 at-risk children positive for islet autoantibodies (AAb+) and 180 age- and HLA-matched autoantibody-negative healthy control children were analyzed. We observed a small, but statistically significant increase in total Treg frequency in children with T1D compared to healthy donors (6.3 ± 1.7% vs. 5.3 ± 1.7%, *P* < 0.0001; Figure 1B). This increase in Treg frequency was, however, not seen in AAb+ children (Figure 1B). Interestingly, the higher frequency of total Tregs in children with T1D was attributable to an increase in the frequency of naive Tregs but not memory Tregs (Figures 1C,D). Correlation analyses revealed that the frequency of total Tregs within the CD4+ T-cell compartment is largely age-independent (Figure 1E). However, the proportion of naive Tregs slowly decreases with age while that of memory Tregs increases (Figures 1F,G). Importantly, the increased frequency of total and naive Tregs in children with T1D was consistently observed in children of all ages (Figures 1E,F). Finally, a strict pairwise comparison with samples from age-matched healthy children processed and analyzed on the same day confirmed our findings (Supplementary Figure 2).

### The Increase of Naive Tregs Is Associated With Progression to Clinical T1D but Not With the Number of Autoantibodies at the Presentation of the Disease

**Figure 2:**
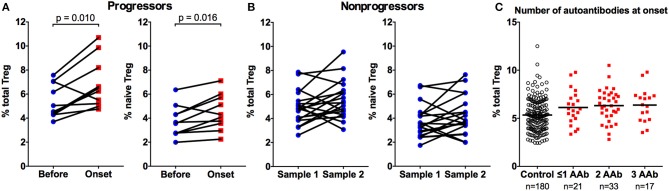
Increase in total and naive Treg frequencies is associated with progression to clinical T1D. Two samples from ten autoantibody-positive children that progressed to T1D were analyzed 3–30 (mean 13) months apart for total (**A**, left) and naive (**A**, right) CD4+CD25+CD127low Treg frequencies. Two samples from 19 autoantibody-positive children that did not progress to T1D were analyzed as a control 3-33 (mean 18) months apart for total (**B**, left) and naive (**B**, right) Treg frequencies. *P* values from paired *t*-tests are indicated. The frequencies of total Tregs in children stratified by the number of biochemical autoantibodies (IAA, GADA, or IA2A) at disease onset **(C)**.

Ten autoantibody-positive children that we analyzed developed clinical T1D during our sample collection period. When we compared the Treg frequencies at the presentation of the disease to the sample analyzed before the diagnosis (mean 13, range 3–30 months earlier), we could clearly demonstrate an increase in total and naive Treg frequencies during this period (Figure 2A). This finding supports the notion that the increase in Treg frequency is not a feature of early islet autoimmunity but rather a phenomenon associated with disease progression. As a control, we analyzed two longitudinal samples (mean 18, range 3–33 months apart) from 19 AAb+ children that did not progress to T1D during our study. No increases in total or naive Treg frequencies were observed between these paired samples (Figure 2B). We further analyzed whether the increase in the frequency of Tregs is associated with the islet autoantibody status at the diagnosis of the disease. For this, we stratified the children with T1D into three groups based on their positivity for one or more biochemical autoantibodies tested (IAA, GADA, and IA-2A). However, no differences between the groups were observed (Figure 2C).

### FOXP3 and HELIOS Stainings Reveal Additional Changes in the Memory Treg Compartment in Children With Newly Diagnosed T1D

**Figure 3:**
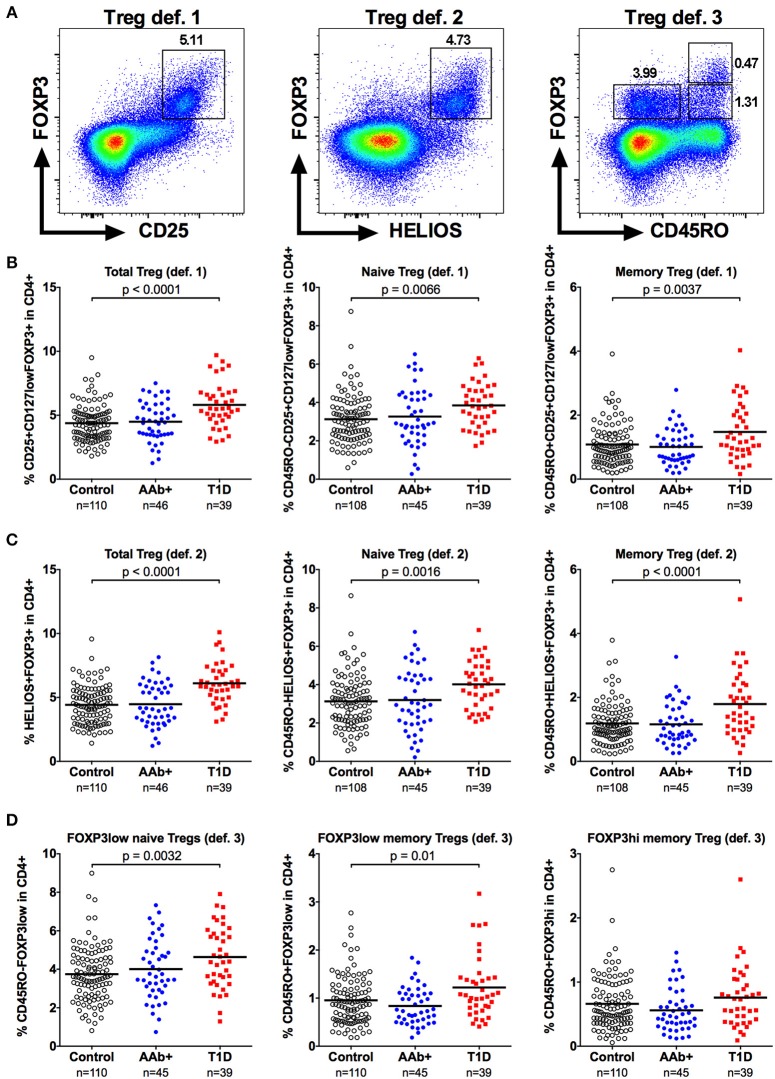
Increased frequencies of FOXP3+ naive and memory Tregs in children with newly diagnosed T1D by different strategies to define Tregs. Tregs were defined either as CD4+CD25+CD127lowFOXP3+ (definition 1; **A**, left), CD4+HELIOS+FOXP3+ (definition 2; **A**, middle) or as CD4+CD45RO-FOXP3low, CD4+CD45RO+FOXP3low and CD4+CD45RO+FOXP3hi (definition 3; **A**, right). Frequencies of definition 1 **(B)**, definition 2 **(C)** and definition 3 **(D)** Tregs in control, AAb+ and T1D groups.

In order to validate our observations, we performed additional analyses utilizing the most specific markers to identify Tregs, the transcription factors FOXP3 and HELIOS. We first employed two commonly used gating approaches to define Tregs: (a) CD4+CD25+CD127lowFOPX3+ (definition 1) and b) CD4+HELIOS+FOXP3+ (definition 2) (23) that were further divided into naive (CD45RO-) and memory (CD45RO+) subsets (Figure 3A and Supplementary Figure 1). The Treg frequencies observed with both of these definitions strongly correlated with the frequencies of CD4+CD25+CD127low cells (Figure 1B) analyzed in parallel (Supplementary Figure 2). Consequently, they permitted us to confirm the increased frequencies of total and naive Tregs in children with T1D (Figures 3B,C and Supplementary Figure 2). Interestingly, using these definitions we also observed a subtle increase in the memory Treg compartment (Figures 3B,C) that was not readily apparent when using the CD4+CD25+CD127low definition for Tregs (Figure 1D). In order to further investigate this phenomenon, we analyzed the Tregs using a third approach (definition 3) originally described by Miyara et al. (25), where FOXP3+ T cells are divided into FOXP3lowCD45RO- naive Treg, FOXP3hiCD45RO+ memory Treg and to non-suppressive FOXP3lowCD45RO+ memory Treg subsets (Figure 3A). This gating strategy once again confirmed the increased frequency of naive Tregs in children with T1D (Figure 3D). Importantly, we could also establish that the increase in FOXP3+ memory Tregs observed with definitions 1 and 2 (Figures 3B,C) was due to an increase in the non-suppressive FOXP3low fraction whereas the FOXP3hi fraction appeared unaltered in children with T1D (Figure 3D and Supplementary Figure 2).

**Figure 4:**
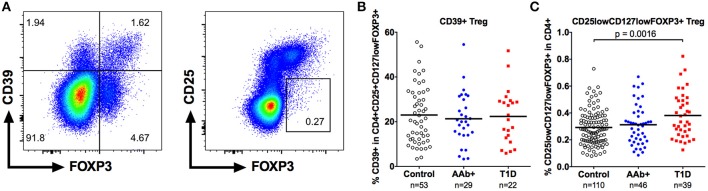
Increased frequency of CD25lowCD127low Tregs but not of CD39+ Tregs in children with newly diagnosed T1D. A subset of CD4+CD25+CD127lowFOXP3 Tregs expresses CD39 (**A**, left). A minor population of FOXP3+ cells is both CD25low and CD127low (**A**, right). Frequencies of CD39+ **(B)** and CD25lowCD127lowFOXP3+ **(C)** Tregs in control, AAb+, and T1D groups.

We also examined the frequency of CD39+ Tregs (Figure 4A) in our cohort. CD39 is exclusively expressed on highly suppressive memory Tregs that are critical in suppressing Th17-type responses, and the frequency of CD39+ Tregs frequency has been reported to be diminished in patients with multiple sclerosis (26, 27). However, the frequency of CD39+ Tregs was not altered in children with T1D or AAb+ children (Figure 4B and Supplementary Figure 3).

Finally, we analyzed the frequency of the minor population of CD25lowCD127lowFOXP3+ Tregs (Figure 4A) that has recently been shown to be increased in peripheral blood of patients with autoimmune diseases, such as systemic lupus erythematosus (SLE) and T1D (11). The frequency of CD25lowCD127lowFOXP3+ Tregs within the CD4+ compartment was slightly elevated in children with T1D (0.38 ± 0.17% vs. 0.29 ± 0.13% in healthy children, *P* < 0.01) but not in autoantibody-positive children (Figure 4C and Supplementary Figure 3).

### The Increased Frequency of Naive Tregs in Children With Newly Diagnosed T1D Does Not Result From Increased Thymic Output or Homeostatic Proliferation

**Figure 5:**
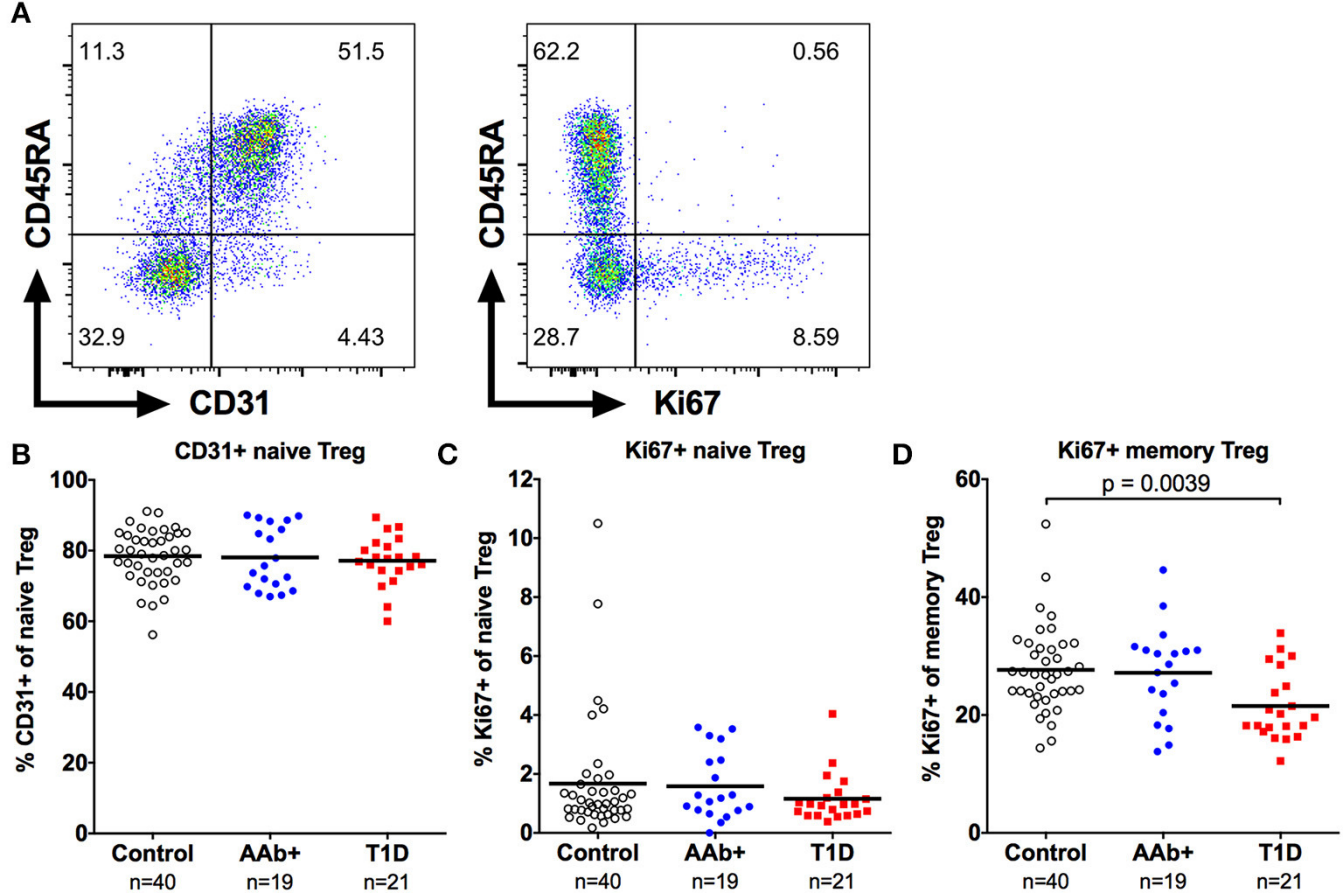
Decreased frequency of proliferating memory Tregs in children with newly diagnosed T1D. Representative examples of CD31 (**A**, left) and Ki67 (**A**, right) expression on CD4+CD25+CD127lowFOXP3+ Tregs. Frequencies of CD31+ naive Tregs **(B)**, and Ki67+ naive **(C)** and memory **(D)** Tregs in control, AAb+ and T1D groups.

The increase in naive Treg frequency in children with newly diagnosed T1D could result either from an enhanced thymic output or an increase in homeostatic proliferation of naive Tregs. To address these possibilities, we analyzed the expression of CD31, which is preferentially expressed by recent thymic emigrant T cells (35), and the proliferation marker Ki67 on Tregs (Figure 5A). No increase in the frequency of CD31+ cells within the naive Treg compartment was observed in children with newly diagnosed T1D (Figure 5B). Moreover, the frequency of proliferating Ki67+ cells within the naive Treg compartment was low, and not altered in children with T1D, even when the CD31+ and CD31- naive Treg subsets were separately analyzed (Figure 5C and Supplementary Figure 3). Interestingly, the frequency of proliferating Ki67+ cells within the memory Treg compartment, and more specifically within the FOXP3hiCD45RO+ memory Treg subset, was lower in children with T1D (Figure 5D and Supplementary Figure 3), providing further evidence of alterations in the memory Treg compartment in T1D. This phenomenon appears to be specific to the Treg compartment as no changes in the frequency of proliferating Ki67+ conventional CD4+CD25- memory CD4+ T cells (Teff) were observed (Supplementary Figure 3). However, Ki67+ memory Treg and Ki67+ memory Teff frequencies correlate strongly (Supplementary Figure 3). Therefore, it is also possible that the decrease in proliferating memory Tregs in children with T1D reflects a more global alteration in the T cell compartment, but this defect is only apparent in the highly proliferative memory Tregs, especially the FOXP3hiCD45RO+ memory Treg subset.

### The Frequencies of CCR6-CXCR3+ Th1-Type Regulatory and Effector T Cells as Well as CXCL10 Plasma Levels Are Increased in Children With Newly Diagnosed T1D

**Figure 6:**
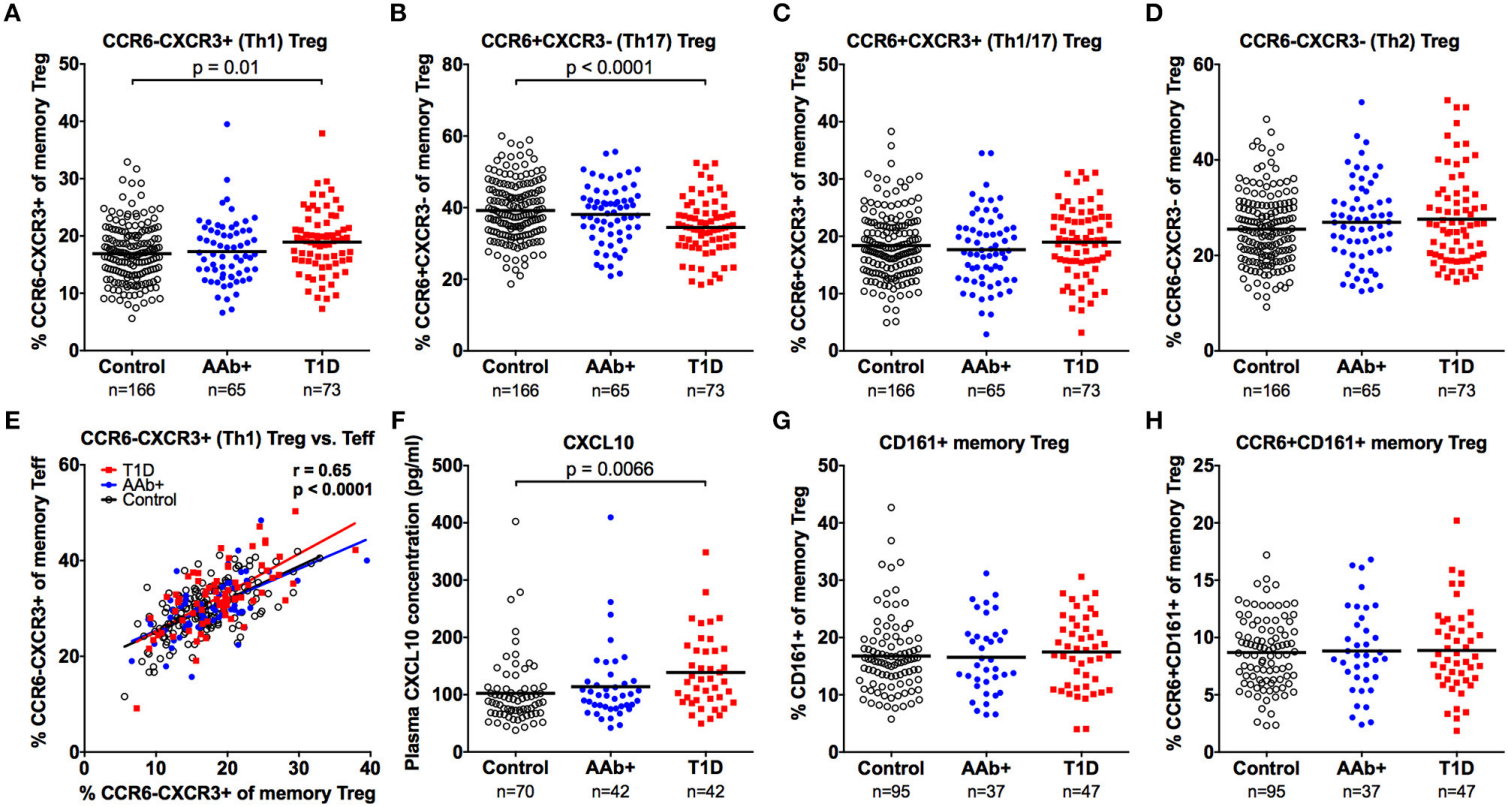
Increased frequency of CXCR3+ memory Tregs and increased plasma CXCL10 levels in children with newly diagnosed T1D. Frequencies of CCR6-CXCR3+ **(A)**, CCR6+CXCR3- **(B)**, CCR6+CXCR3+ **(C)**, and CCR6-CXCR3- **(D)** memory Tregs in control, AAb+ and T1D groups. Linear regression lines of CCR6-CXCR3+ memory Tregs against Teffs were calculated for the control (black lines), AAb+ (blue lines) and T1D (red lines) groups **(E)**. Correlation was calculated by pooling all samples analyzed and is expressed together with the *P* value next to the plot. Plasma CXCL10 levels **(F)**, and CD161+ **(G)** and CCR6+CD161+ **(H)** memory Treg frequencies in control, AAb+ and T1D groups.

A proportion of memory Tregs display characteristics associated with the Th1, Th2, Th17, or Tfh lineages that can be identified based on the expression of chemokine receptors as well as lineage-specific transcription factors or cytokines (28–32). To address this heterogeneity in our study cohorts, we first assessed the expression of the chemokine receptors CCR6 and CXCR3 on memory Tregs (Supplementary Figure 4). Based on the expression of these markers, memory CD4+ T cells can be subdivided into subsets that are enriched for Th2 (CCR6-CXCR3-), Th1 (CCR6-CXCR3+), Th17 (CCR6+CXCR3-), and Th1/17 (CCR6+CXCR3+) cells both within the Teff (36–38) and Treg (30) compartments. We observed that the proportion of Th1-type (CCR6-CXCR3+) Tregs was increased and that of Th17-type (CCR6+CXCR3-) Tregs was decreased in children with T1D (Figures 6A–D and Supplementary Figure 4). However, this phenomenon was not specific to the Treg compartment, as the same changes could be observed in the CD4+CD25- memory Teff compartment of children with T1D (Supplementary Figure 4). Moreover, the phenotype of memory Tregs and memory Teffs appeared to correlate strongly within an individual (Figure 6E). Interestingly, we could also demonstrate an increased concentration of CXCL10, the chemokine ligand for CXCR3, in plasma samples from children with newly diagnosed T1D but not in AAb+ children (Figure 6F and Supplementary Figure 4).

We also examined the frequencies of CD161+ and CCR6+CD161+ memory Tregs (Supplementary Figure 4), two populations that have previously been shown to contain Tregs with proinflammatory potential (33, 34). No differences in these subset frequencies were observed between the study groups (Figures 6G,H and Supplementary Figure 4).

### The Frequency of Circulating CXCR5+FOXP3+ T Follicular Regulatory Cells Is Not Altered in Children With Newly Diagnosed T1D

**Figure 7:**
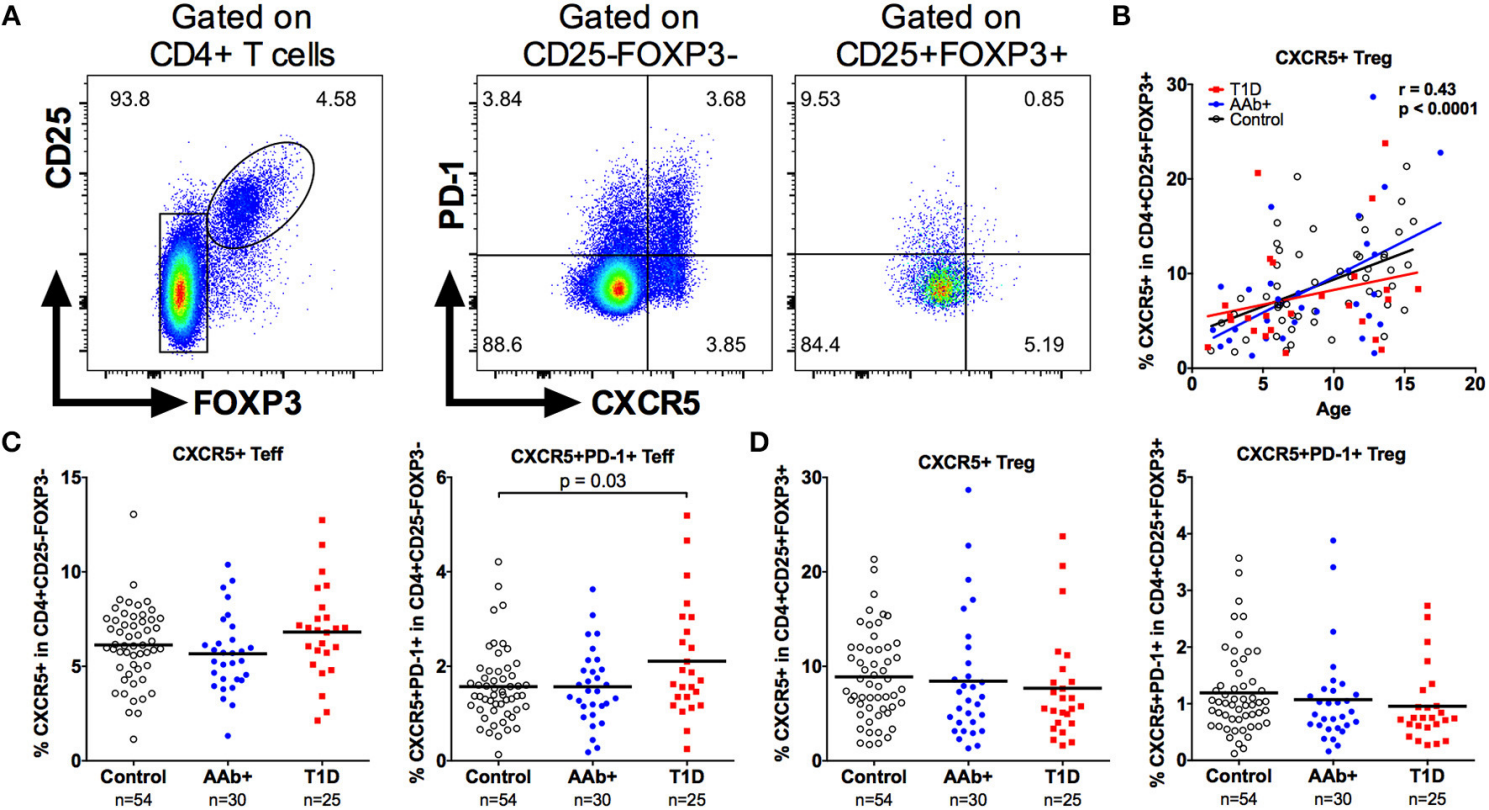
Increased frequency of circulating Tfh but not Tfr cells in children with newly diagnosed T1D. Tfh cells were defined as CD4+CD25-FOXP3-CXCR5+ and Tfr cells as CD4+CD25+FOXP3+CXCR5+ cells **(A)**. Linear regression lines for CXCR5+ Treg frequencies against age were calculated for the control (black lines), AAb+ (blue lines) and T1D (red lines) groups **(B)**. Correlation was calculated by pooling all samples analyzed and is expressed together with the *P* value next to the plot. Frequencies of CXCR5+ and CXCR5+PD-1+ Teffs (Tfh; **C**) and frequencies of CXCR5+ and CXCR5+PD-1+ Tregs (Tfr; **D**) in control, AAb+, and T1D groups.

A subset of circulating Tregs expresses CXCR5 and is thought to represent circulating T follicular regulatory cells (Tfr), although the exact biological function of CXCR5+ Tregs is currently unclear (32). Importantly, alterations in the frequencies of circulating Tfr have recently been reported in patients with multiple sclerosis and SLE (32, 39). Using a similar gating strategy, we confirm that a subset of CD4+CD25+FOXP3+ Tregs expresses CXCR5 (Figure 7A). Moreover, consistent with previous investigations (32), CXCR5+ Tregs express lower levels of CXCR5, PD-1, and CD45RO compared to conventional circulating CXCR5+ follicular helper T cells (Tfh; Supplementary Figure 5). The frequency of CXCR5+ Tregs also increases strongly with age (Figure 7B). In accordance with previous reports (40–42), we observed an increased frequency of CXCR5+PD-1+ circulating Tfh in children with T1D, especially in those positive for multiple autoantibodies (Figure 7C and Supplementary Figure 5). However, the frequencies of either CXCR5+ or CXCR5+PD-1+ Tregs were not altered in children with T1D or in AAb+ children (Figure 7D and Supplementary Figure 5).

### The Frequency of IFN-γ -Producing Memory Tregs Is Decreased in Children With Newly Diagnosed T1D

**Figure 8:**
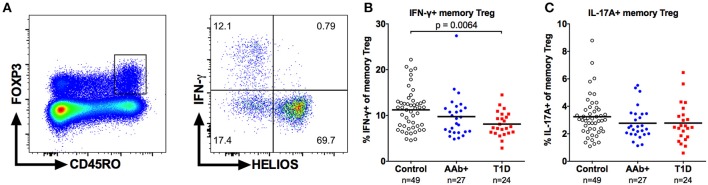
Decreased frequency of IFN-γ-producing memory Tregs in children with newly diagnosed T1D. Representative example of IFN-γ production by CD4+CD25+FOXP3+HELIOS-CD45RO+ memory Tregs **(A)**. Frequencies of IFN-γ- **(B)** and IL-17A-producing **(C)** memory Tregs in control, AAb+ and T1D groups.

Finally, to investigate the capacity of Tregs to produce proinflammatory cytokines, we stimulated PBMCs with PMA and ionomycin and analyzed the production of IFN-γ and IL-17A by FOXP3+ memory Tregs (Figure 8A). In accordance with previous reports (17, 41), the cytokine-producing Tregs were exclusively contained in the HELIOS-negative subset of memory Tregs (Figure 8A). Importantly, the frequency of IFN-γ-producing memory Tregs was reduced in children with T1D but not in AAb+ children compared to healthy controls (Figure 8B and Supplementary Figure 6). No differences in the frequency of IL-17A-producing memory Tregs were observed between the different study groups (Figure 8C). The decrease in IFN-γ-producing cells appeared to be specific for the Treg compartment, as no differences in the frequencies of IFN-γ or IL-17A-producing memory Teffs were observed between the study groups (Supplementary Figure 6).

## Discussion

Regulatory T cell dysfunction has long been suspected to play a crucial role in the pathogenesis of T1D. Over the years, many studies have assessed whether the frequency or phenotype of peripheral blood Tregs is altered in patients with T1D. Here, we add to these reports our current findings using samples from a large and well-controlled natural history cohort of pediatric T1D. We observed multiple subtle changes in the peripheral Treg compartment of children with newly diagnosed T1D but none in autoantibody-positive at-risk children.

Until recently, the general consensus from several studies has been that there is no clear alteration in the frequency of peripheral blood CD4+FOXP3+ Tregs in patients with T1D (13–19), although some studies have reported both elevated (10, 11) and decreased frequencies (12). Here, we demonstrate a subtle increase in the frequency of FOXP3+ Tregs in children with T1D using multiple different approaches to define Tregs. Our results also highlight the importance of examining specific subsets of Tregs, as we can clearly demonstrate that the increase in total Tregs is caused both by an increase in the frequency of naive FOXP3+ Tregs as well by an increase in FOXP3lo memory Tregs. Importantly, both of these findings have previously been reported in patients with T1D (10, 18) as well as in patients with another autoimmune disease, SLE (25, 43). In addition, we confirmed here the results of a recent report (11) that the frequency of the atypical population of CD25lowCD127lowFOXP3+ T cells is increased in patients with T1D. Whether the observed increases in these three Treg subsets are mechanistically linked with each other remains an open question. Moreover, it is possible that additional small subsets could also be altered, given that there may be more than 20 phenotypically distinct subsets within the FOXP3+ Treg compartment (24).

The most striking finding of our study was the increase in the frequency of naive Tregs at the presentation of T1D. Using longitudinal samples, we could confirm that this change was associated with progression to clinical disease. Potential explanations for this phenomenon include an increased thymic output of naive Tregs or their increased proliferation *in vivo*. However, we observed no increase in the expression of CD31, a marker for recent thymic emigrant T cells, or the proliferation marker Ki67 in naive Tregs from children with T1D. An alternative explanation could be a change in the distribution of naive Tregs between blood and lymphoid tissues due to the inflammatory milieu at the manifestation of T1D. In line with this interpretation the increase in naive Treg frequency in patients with SLE was shown to correlate with both the clinical activity of the disease as well as with serum proinflammatory cytokine levels (43).

The increase in FOXP3lo memory Tregs in children with T1D is an interesting finding, since this population has previously been shown to be largely non-suppressive and capable of producing proinflammatory cytokines (10, 25). Therefore, we further analyzed the expression of chemokine receptors and cytokine production within the memory Treg compartment in samples from our clinical cohort. The frequency of CCR6-CXCR3+ (Th1-type) T cells was increased and that of CCR6+CXCR3- (Th17-type) T cells decreased both in the memory Treg and Teff compartments of children with newly diagnosed T1D. Moreover, in accordance with previous reports (44, 45), serum levels of CXCL10, the chemokine ligand for CXCR3, were observed to be increased in children with T1D.

In murine models, CXCR3+ Tregs have been shown to migrate to sites of inflammation in response to CXCL10 and to specifically suppress Th1-type immune responses (29, 31). Similarly, CCR6+ Tregs appear to migrate to inflamed tissues in response to their chemokine ligand CCL20 (28). Therefore, the observed imbalance of CXCR3+ and CCR6+ T cells in children with T1D can indicate disturbances in T-cell migration patterns. The increase in circulating CXCR3-expressing T cells could result from increased systemic CXCL10 levels in patients with T1D, as observed here. The proportional decrease in CCR6+ Tregs, on the other hand, could be a result of increased migration of CCR6+ T cells into inflamed tissues.

Interestingly, using immunohistochemistry, both CXCL10 expression by β-cells and CXCR3 expression on T cells have been observed to be increased in pancreatic islets of patients with T1D (46–48). In murine models of T1D, CXCL10 production by pancreatic β-cells has been shown to recruit pathogenic autoreactive CXCR3+ T cells to pancreatic islets (49, 50). However, CXCR3 expression on Tregs appears also to be crucial in protecting against autoimmune pathology, suggesting both a pathogenic and protective role for CXCR3 in T1D (31). Therefore, the increase in peripheral blood CXCR3+ Tregs observed here could also be associated with an attempt of the immune system to harness Th1-type immunopathology at the manifestation of T1D. Although inhibition of CXCR3 is an attractive therapeutic strategy to prevent the infiltration of autoreactive T cells into pancreatic islets, caution has to be taken with this approach, since it may also inhibit CXCR3+ Tregs that can have a protective effect on autoimmune pathology in human T1D.

Previous studies have suggested that the frequency of Tregs producing either IFN-γ or IL-17A is increased in patients with T1D (10, 17). CD161-expression can be used to identify the subpopulation of memory Tregs with the capacity to produce proinflammatory cytokines (33, 34). However, we observed no increase of CD161+ memory Treg in children with T1D. Importantly, we also did not observe a difference in the frequency of IL-17A-producing memory Tregs and the frequency of IFN-γ-producing memory Tregs was lower in children with T1D. The discrepancy between our current results and earlier reports may be related to differences in the methodological approach or the clinical cohorts analyzed. Notably, the original studies by McClymont et al. and Marwaha et al. (10, 17) demonstrated an increased frequency of IFN-γ- and IL-17A-producing memory Tregs after *in vitro* expansion in a rather small cohort of subjects. In line with our current results, a more recent study analyzing a larger cohort of patients did not observe an increased frequency of IFN-γ-producing memory Tregs directly *ex vivo* (41).

Recent studies by us and others have demonstrated that the frequency of circulating CXCR5+ Tfh cells is increased in patients with T1D (40–42). We corroborated these findings here by demonstrating an increased frequency of circulating CXCR5+PD-1+ Tfh cells in children with T1D. The frequency of CXCR5+ Tregs, presumably reflecting circulating T follicular regulatory cells (Tfrs), however, was not increased in children with T1D. These findings potentially reflect an imbalance between Tfh and Tfr responses in T1D that could result in enhanced B cell autoimmunity. Interestingly, T1D seems also to differ in this aspect from two other autoimmune diseases, multiple sclerosis and SLE, where increased circulating Tfr frequencies have been reported (32, 39).

An important open research question is whether in addition to altered frequencies there are also functional defects within the different Treg subsets in children with T1D. Addressing this key question is, however, technically challenging since the isolation of sufficient numbers of rare Treg subsets for functional assays is largely precluded by the limited volume of blood obtainable from pediatric subjects. Another obvious caveat of our study is that we could only analyze Treg frequency and phenotype in blood samples, where the changes are likely to be minor compared to those in pancreatic lymph nodes (pLNs) and inflamed islets. In the NOD mouse model, there appears to be an increased frequency of Tregs in pLNs but a decreased frequency in inflamed islets of newly diabetic mice (5), although the frequency of Tregs in pLNs possibly decreases after disease onset (51). Moreover, a seminal study by Ferraro et al. demonstrated a decreased frequency of FOXP3+ Tregs with concomitant increase in Th17 cells in pLNs of patients with long-standing T1D, which was not accompanied with similar changes in the peripheral blood (16). Although technically challenging, more studies addressing Treg phenotype and function in human pLNs and islets are obviously needed in order to understand the potential contribution of Treg dysfunction to T1D pathogenesis.

In summary, we demonstrate here multiple subtle differences in peripheral blood Treg subsets in children with newly diagnosed T1D that are not observable in autoantibody-positive at-risk children. This strongly suggests that peripheral blood Treg alterations are either associated with later stages of the autoimmune progression to clinical T1D or are secondary to the islet inflammation or metabolic dysfunction at the presentation of the disease. Our current work increases the understanding of Treg dysfunction during the natural history of T1D, which can be important both for improving current immunotherapeutic strategies to prevent or treat T1D through enhancing Treg functionality, as well as for the development of better biomarkers to monitor disease progression and immunotherapeutic efficacy.

## Ethics Statement

This study was carried out in accordance with the recommendations of the ethical committee of Turku University Hospital with written informed consent from all subjects. All subjects gave written informed consent in accordance with the Declaration of Helsinki. The protocol was approved by the ethical committee of Turku University Hospital.

## Author Contributions

TV, AG, E-LI, and IE performed the experiments. KN-S and JT provided the clinical samples. RV and MK were responsible for the analyses of diabetes-associated autoantibodies. JI was responsible for the HLA screening of the study children. AG and TK analyzed the data and drafted the manuscript. All authors contributed to the final version of the manuscript. TK is the guarantor of this work and, as such, had full access to all of the data in the study and takes responsibility for the integrity of the data and the accuracy of the data analysis.

### Conflict of Interest Statement

The authors declare that the research was conducted in the absence of any commercial or financial relationships that could be construed as a potential conflict of interest.
