# Supplementary material for: FOXP3+ Regulatory T Cell Compartment Is Altered in Children With Newly Diagnosed Type 1 Diabetes but Not in Autoantibody-Positive at-Risk Children
Source: Front Immunol. 2019 Jan 22;10:19. doi: 10.3389/fimmu.2019.00019 (PMC6349758; doi:10.3389/fimmu.2019.00019)
Supplement: Supplementary file 1 [file Data_Sheet_1.pdf]

Supplementary Material

FOXP3+ regulatory T cell compartment is altered in children with newly diagnosed type 1 diabetes but not in autoantibody-positive at-risk children

Tyyne Viisanen,[1#] Ahmad M Gazali,[1#] Emmi-Leena Ihantola,[1] Ilse Ekman,[1] Kirsti Näntö-Salonen,[2] Riitta Veijola,[3] Jorma Toppari,[2,4] Mikael Knip,[5,6,7,8] Jorma Ilonen,[9,10] and Tuure Kinnunen[1,11*]

[1]Department of Clinical Microbiology, Institute of Clinical Medicine, University of Eastern Finland, Kuopio, Finland

[2]Department of Pediatrics, Turku University Hospital, Turku, Finland

[3]Department of Pediatrics, Medical Research Center, PEDEGO Research Unit, Oulu University Hospital and University of Oulu, Oulu, Finland

[4]Department of Physiology, Institute of Biomedicine, University of Turku, Turku, Finland

[5]Tampere Center for Child Health Research, Tampere University Hospital, Tampere, Finland

[6]Children's Hospital, University of Helsinki and Helsinki University Hospital, Helsinki, Finland

[7]Research Programs Unit, Diabetes and Obesity, University of Helsinki, Helsinki, Finland

[8]Folkhälsan Research Center, Helsinki, Finland

[9]Immunogenetics Laboratory, Institute of Biomedicine, University of Turku, Turku, Finland

[10]Clinical Microbiology, Turku University Hospital, Turku, Finland

[11]Eastern Finland Laboratory Centre (ISLAB), Kuopio, Finland

[#]These authors contributed equally to the study

*** Correspondence:**
Dr. Tuure Kinnunen
tuure.kinnunen@uef.fi

Supplementary Table 1. Fluorescent monoclonal antibodies used in the study

**Staining panel**	**Antibody**	**Manufacturer**	**Clone**
**Treg panel 1** (Figures 1, 2 and 6)	CD4 APC-H7	BD Biosciences	RPA-T4
	CD25 PE-Cy7	BioLegend	BC96
	CCR6 PE	BioLegend	G034E3
	CXCR3 A647	BioLegend	G025H7
	CD127 PerCp-Cy5.5	BioLegend	A019D5
	CD45RA FITC	BioLegend	HI100
**Treg panel 2** (Figures 3 and 4)	CD3 APC-H7	BD Biosciences	SK-7
	CD4 BV510	BioLegend	RPA-T4
	CD45RO BV421	BioLegend	UCHL1
	CD39 PE-Cy7	BioLegend	A1
	CD25 PE	Miltenyi Biotec	4E3
	CD127 PerCp-Cy5.5	BioLegend	A019D5
	FOXP3 A488	BioLegend	259D
	HELIOS A647	BioLegend	22F6

**Treg panel 3 (Figure 5)**	CD3 APC-H7	BD Biosciences	SK-7
	CD4 BV510	BioLegend	RPA-T4
	Ki-67 BV421	BioLegend	Ki-67
	CD25 PE-Cy7	BioLegend	BC96
	CD127 PerCp-Cy5.5	BioLegend	A019D5
	FOXP3 A647	BioLegend	259D
	CD45RA FITC	BioLegend	HI100
	CD31 PE	Miltenyi Biotec	AC128
**Treg panel 4 (Figure 6)**	CD3 APC-H7	BD Biosciences	SK-7
	CD4 BV510	BioLegend	RPA-T4
	CD45RO BV421	BioLegend	UCHL1
	CD25 PE-Cy7	BioLegend	BC96
	CD127 PerCp-Cy5.5	BioLegend	A019D5
	FOXP3 A488	BioLegend	259D
	CD161 A647	BioLegend	HP-3G10
	CCR6 PE	BioLegend	G034E3

**Treg panel 5 (Figure 7)**	CD3 APC-H7	BD Biosciences	SK-7
	CD4 BV510	BioLegend	RPA-T4
	CD45RO BV421	BioLegend	UCHL1
	CD25 PE-Cy7	BioLegend	BC96
	CD127 PerCp-Cy5.5	BioLegend	A019D5
	FOXP3 A488	BioLegend	259D
	PD-1 A647	BioLegend	EH12.2H7
	CXCR5 PE	BioLegend	J252D4
**Treg panel 6 (Figure 8)**	CD3 APC-H7	BD Biosciences	SK-7
	CD4 BV510	BioLegend	RPA-T4
	CD45RO BV421	BioLegend	UCHL1
	CD25 PE-Cy7	BioLegend	BC96
	CD127 PerCp-Cy5.5	BioLegend	A019D5
	FOXP3 A488	BioLegend	259D
	HELIOS A647	BioLegend	22F6
	IFN-γ or IL-17A PE	BioLegend	4S.B3 or BL168

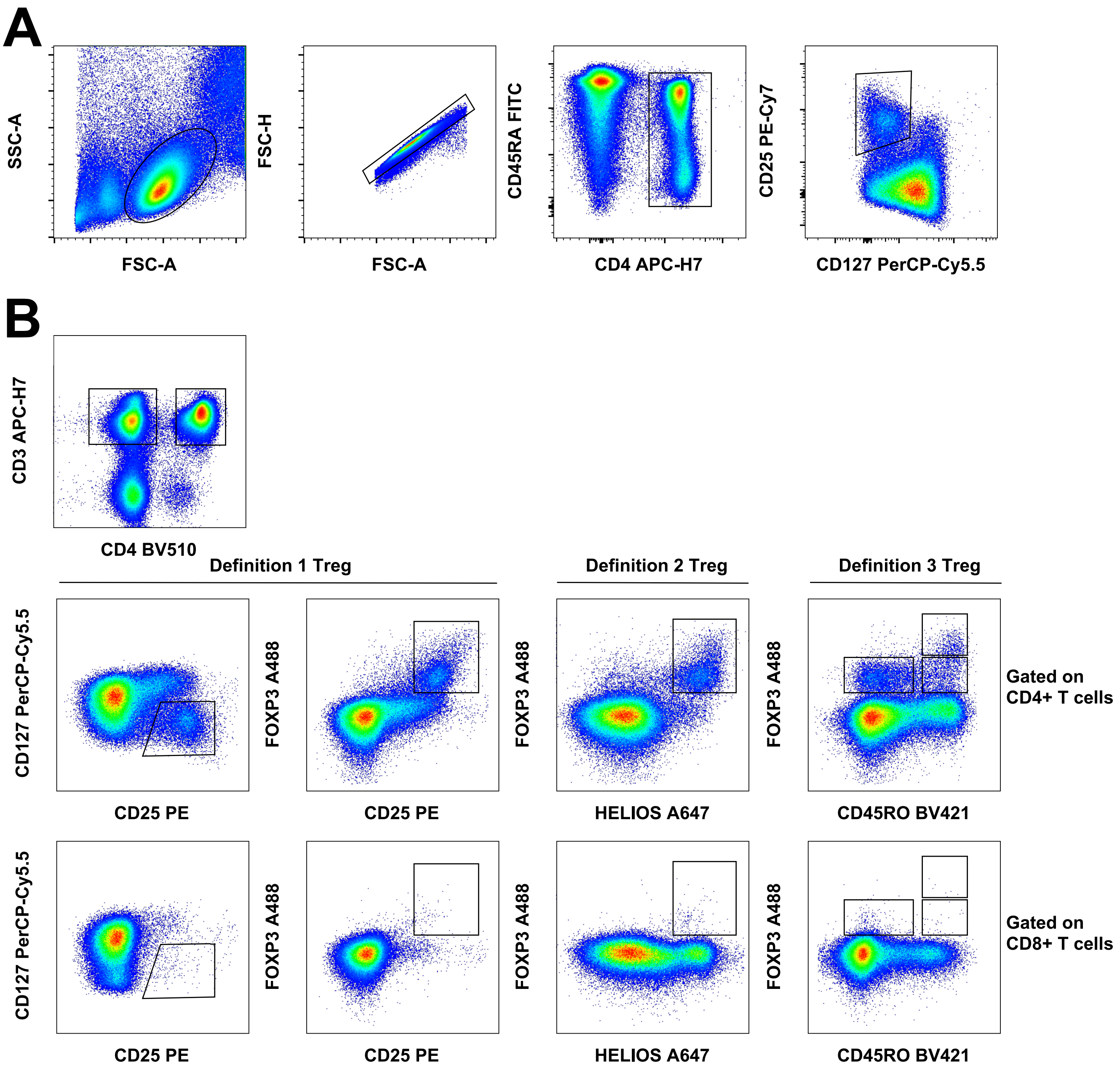

Supplementary Figure 1. Gating strategies to define Tregs. Lymphocytes were first gated based on FSC and SSC properties, followed by elimination of doublets through FSC-A and FSC-H gating. CD4+CD25+CD127low Tregs (**A**, related to Figure 1) were gated as shown. For gating of definition 1, 2 and 3 Tregs (**B**, related to Figure 3), cells were first gated as CD3+CD4+ followed by gating for CD25+CD127low and FOXP3+ (def. 1), HELIOS+FOXP3+ (def. 2) and CD45RO and FOXP3 (def. 3). Gates were set based on FMO controls. CD3+CD4- (CD8+ T cells) within the same samples were also used as internal biological gating controls, as they lack FOXP3 and CD25 expression (**B**).

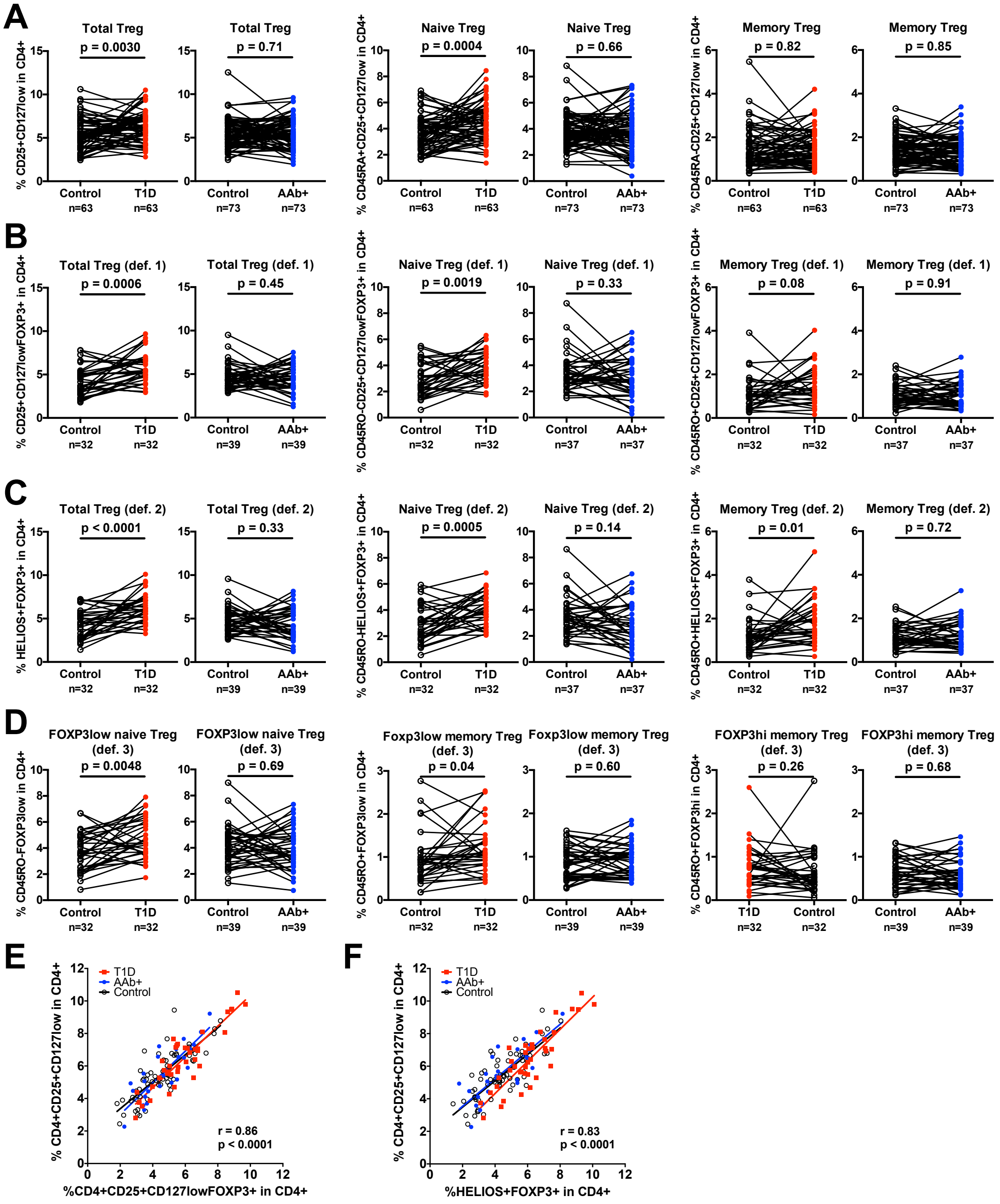

Supplementary Figure 2. Pairwise analyses of Treg frequencies. CD4+CD25+CD127low (**A**, related to Figure 1), CD4+CD25+CD127lowFOXP3+ (**B**, related to Figure 3), HELIOS+FOXP3+ (**C**, related to Figure 3), and CD45RO-FOXP3low, CD45RO+FOXP3low and CD45RO+FOXP3hi (**D**, related to Figure 3) Treg frequencies in samples from children with newly diagnosed T1D or autoantibody-positive children were compared to the frequencies in samples from age-matched healthy children (control) processed and analyzed in parallel. *P* values from paired t-tests are indicated. Linear regression lines for CD4+CD25+CD127lowFOXP3+ (**E**) and CD4+HELIOS+FOXP3+ (**F**) against CD4+CD25+CD127low Treg frequencies were calculated for the control (black lines), AAb+ (blue lines) and T1D (red lines) groups. Correlation was calculated by pooling all samples analyzed and is expressed together with *P* values next to the individual plots.

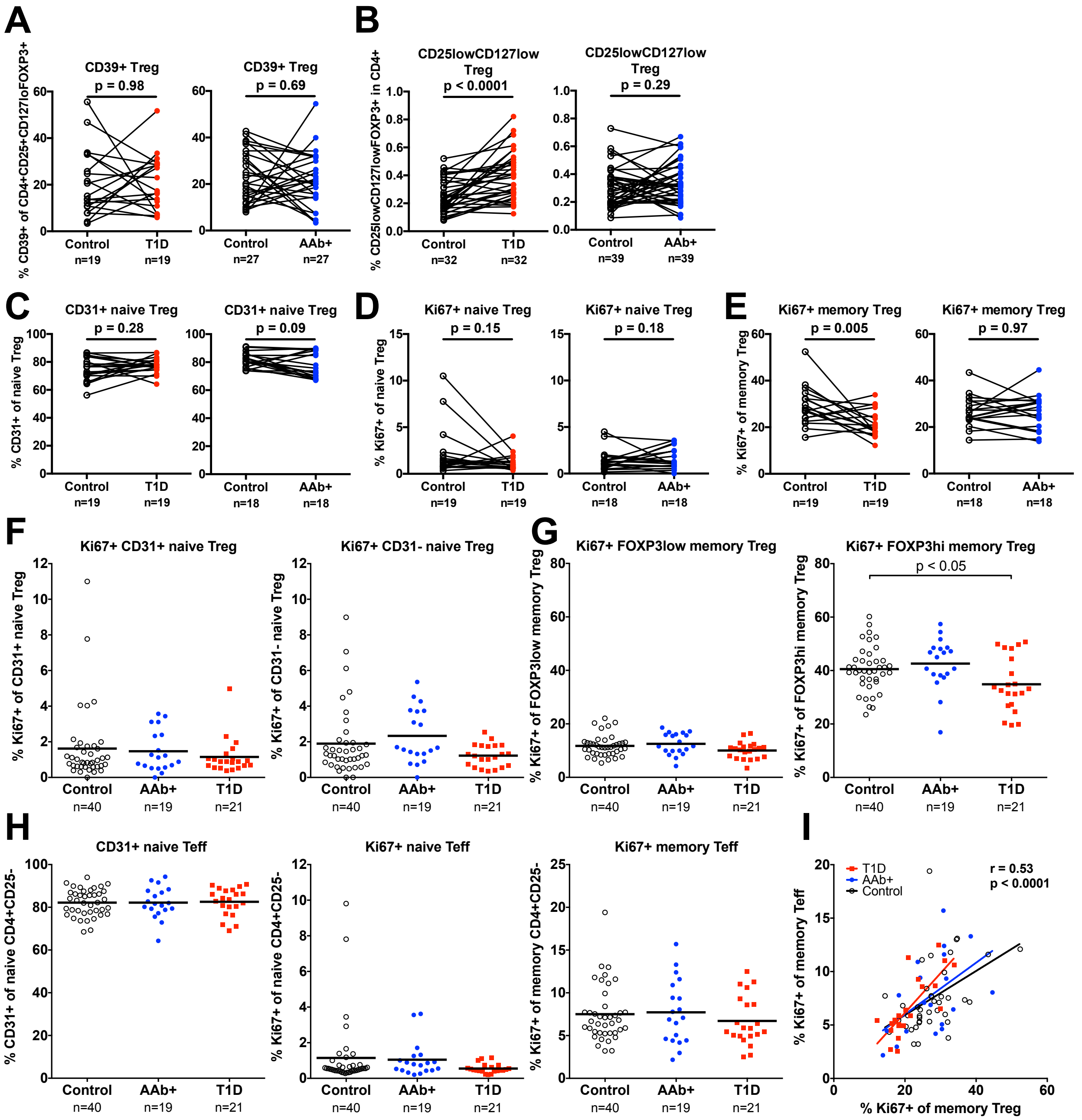

Supplementary Figure 3. Pairwise analyses of CD39+ Treg (**A**, related to Figure 4), CD25lowCD127low Treg (**B**, related to Figure 4), CD31+ naive Treg (**C**, related to Figure 5), Ki67+ naive Treg (**D**, related to Figure 5) and Ki67+ memory Treg (**E**, related to Figure 5) frequencies in samples from children with newly diagnosed T1D or autoantibody-positive children compared to the frequencies in samples from age-matched healthy children (control) processed and analyzed in parallel. *P* values from paired t-tests are indicated. Frequencies of Ki67+CD31+ (**F**, left) and Ki67+CD31- (**F**, right) naive Tregs, frequencies of Ki67+FOXP3low (**G**, left) and Ki67+FOXP3hi (**G**, right) memory Tregs, and frequencies of CD31+ naive CD4+CD25- Teff (**H**, left), Ki67+ naive Teff (**H**, middle) and Ki67+ memory Teff (**H**, right) in control, AAb+ and T1D groups. Linear regression lines for Ki67+ memory Treg against Ki67+ memory Teff frequencies were calculated for the control (black lines), AAb+ (blue lines) and T1D (red lines) groups (**I**). Correlation was calculated by pooling all samples analyzed and is expressed together with the *P* value on the plot.

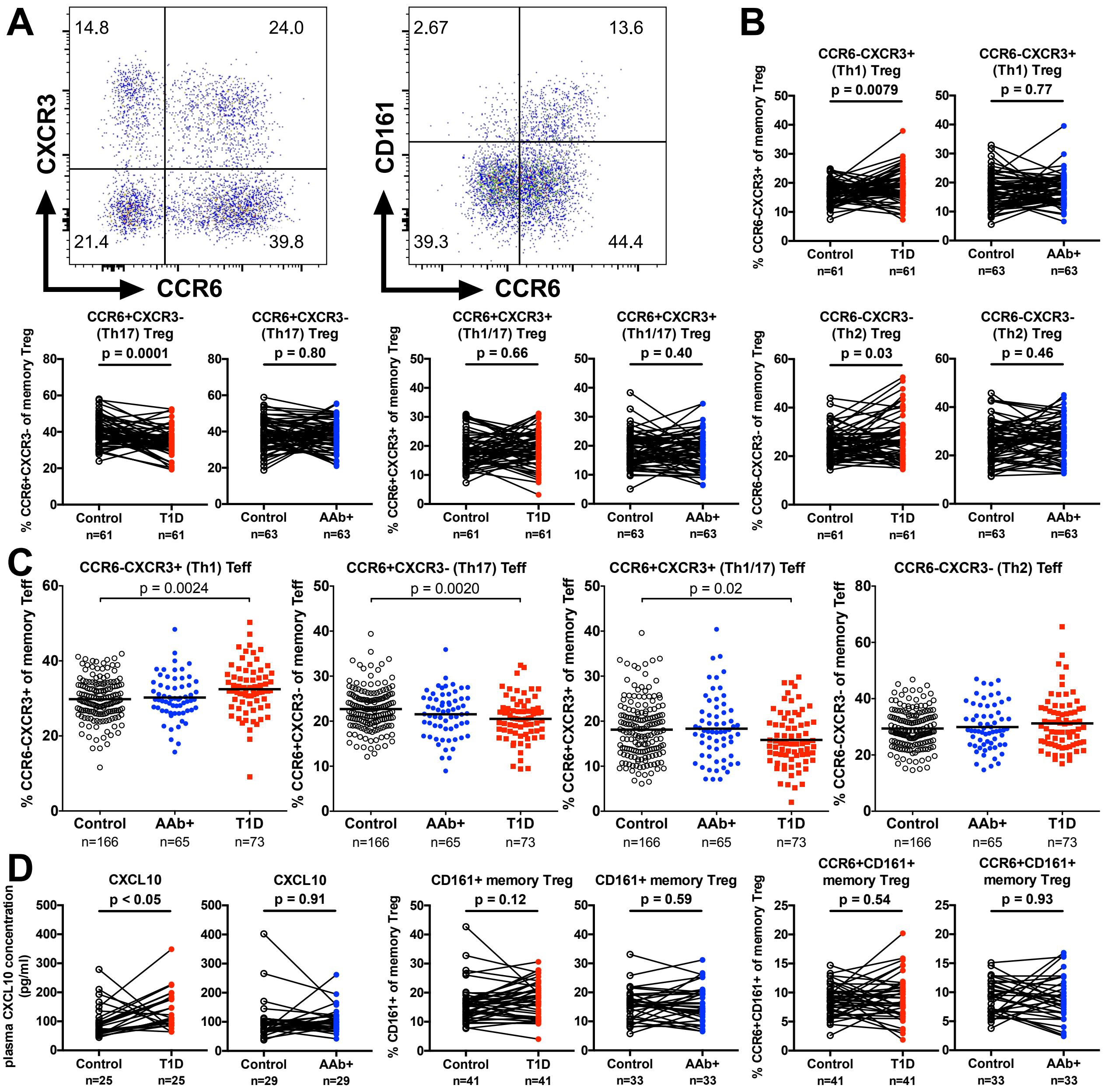

Supplementary Figure 4. Representative examples of CCR6 and CXCR3 (**A**, left) and CCR6 and CD161 (**A**, right) expression on CD4+CD25+CD127lowFOXP3+CD45RA- memory Tregs. Pairwise analyses of CCR6-CXCR3+, CCR6+CXCR3-, CCR6+CXCR3+ and CCR6-CXCR3- memory Tregs in samples from children with newly diagnosed T1D or autoantibody-positive children compared to the frequencies in samples from age-matched healthy children (control) processed and analyzed in parallel (**B**, related to Figure 6). *P* values from paired t-tests are indicated. Frequencies of CCR6-CXCR3+, CCR6+CXCR3-, CCR6+CXCR3+ and CCR6-CXCR3- memory Teff in control, AAb+ and T1D groups (**C**). Pairwise analyses of plasma CXCL10 levels, and CD161+ and CCR6+CD161+ memory Treg frequencies (**D**).

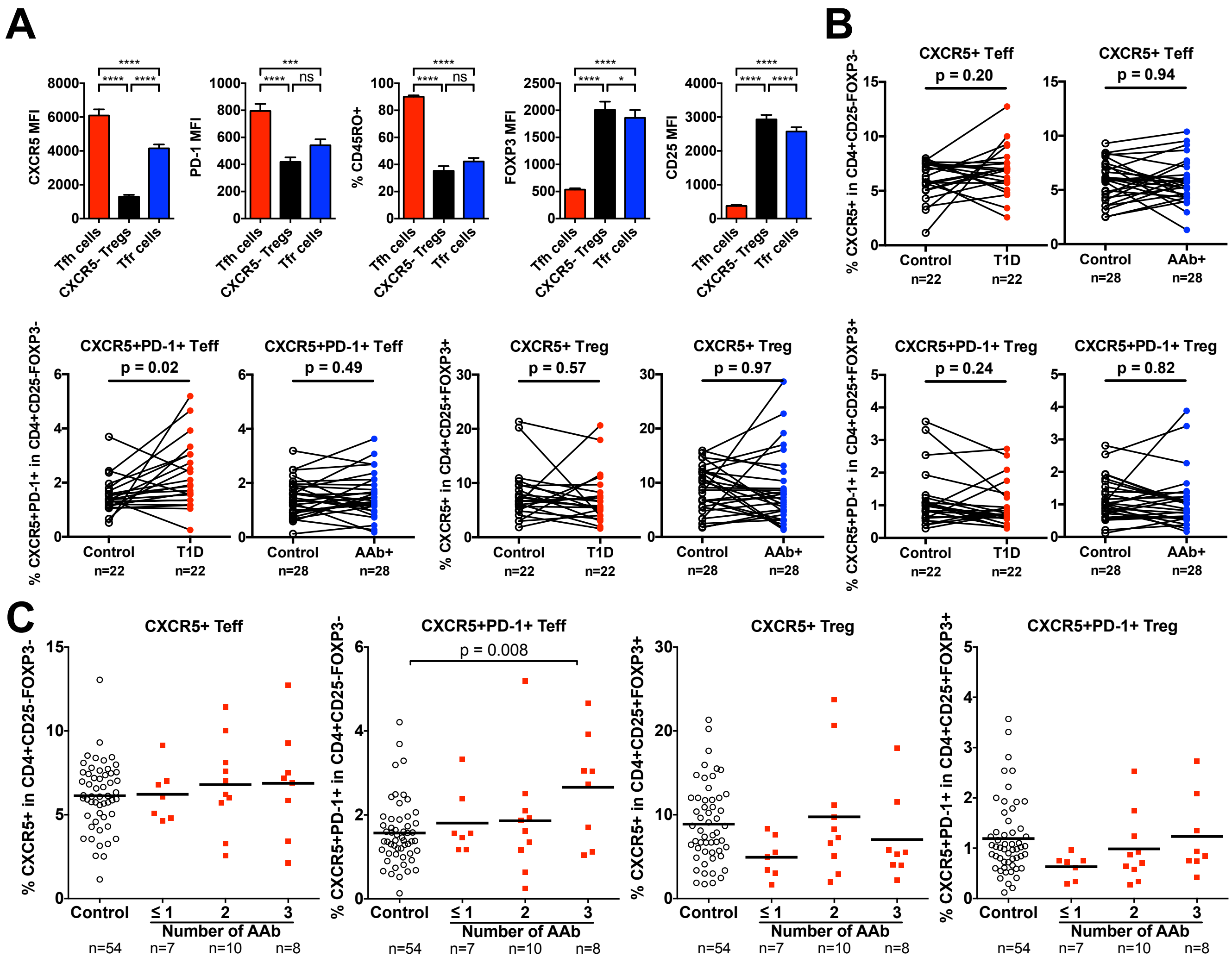

Supplementary Figure 5. Expression of CXCR5, PD-1, CD45RO, FOXP3 and CD25 on peripheral blood Tfh cells (CD4+CD25-FOXP3-CXCR5+), CXCR5- Tregs (CD4+CD25+FOXP3+CXCR5-) and Tfr cells (CD4+CD25+FOXP3+CXCR5+) in healthy control children (**A**; n=12, *P<0.05, ***P<0.001 and ****P<0.0001; one-way ANOVA with Tukey's multiple comparison test). Pairwise analyses of CXCR5+ and CXCR5+PD-1+ memory Teffs (Tfh) and CXCR5+ and CXCR5+PD-1+ memory Tregs (Tfrs) in samples from children with newly diagnosed T1D or autoantibody-positive children compared to frequencies in samples from age-matched healthy children (control) processed and analyzed in parallel (**B**, related to Figure 7). *P* values from paired t-tests are indicated. Frequencies of CXCR5+ and CXCR5+PD-1+ memory Teffs (Tfh) and CXCR5+ and CXCR5+PD-1+ memory Tregs (Tfrs) in children with newly diagnosed T1D stratified into subgroups based on the number of biochemical autoantibodies (IAA, GADA and IA2A) at disease onset (**C**).

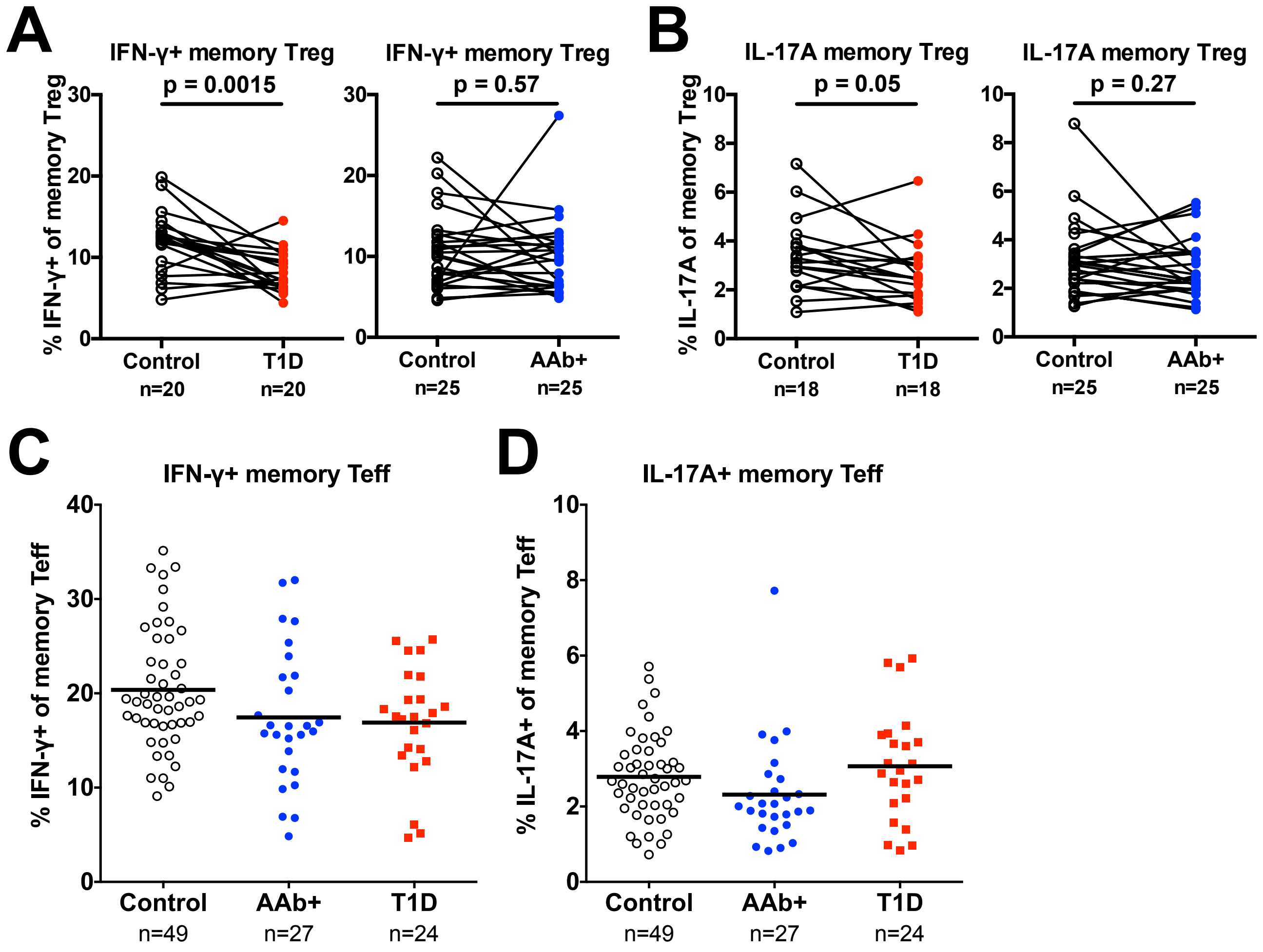

Supplementary Figure 6. Pairwise analyses of IFN-γ-producing (**A**) and IL-17A-producing (**B**) memory Tregs in samples from children with newly diagnosed T1D or autoantibody-positive children compared to the frequencies in samples from age-matched healthy children (control) processed and analyzed in parallel (related to Figure 8). *P* values from paired t-tests are indicated. Frequencies of IFN-γ-producing (**C**) and IL-17A-producing (**D**) memory Teff in control, AAb+ and T1D groups.
